# Insulin Secretory Actions of Ethanol Extract of *Eucalyptus citriodora* Leaf, including Plasma DPP-IV and GLP-1 Levels in High-Fat-Fed Rats, as Well as Characterization of Biologically Effective Phytoconstituents

**DOI:** 10.3390/metabo12080757

**Published:** 2022-08-17

**Authors:** Prawej Ansari, Samara T. Choudhury, Yasser H. A. Abdel-Wahab

**Affiliations:** 1 Department of Pharmacy, School of Pharmacy and Public Health, Independent University, Bangladesh (IUB), Dhaka 1229, Bangladesh; 2 School of Biomedical Sciences, Ulster University, Coleraine BT52 1SA, UK

**Keywords:** insulin, medicinal plants, diabetes, glucose, phytoconstituents

## Abstract

Due to the numerous adverse effects of synthetic drugs, researchers are currently studying traditional medicinal plants to find alternatives for diabetes treatment. *Eucalyptus citriodora* is known to be used as a remedy for various illnesses, including diabetes. This study aimed to explore the effects of ethanol extract of *Eucalyptus citriodora* (EEEC) on in vitro and in vivo systems, including the mechanism/s of action. The methodology used involved the measurement of insulin secretion from clonal pancreatic β-cells, BRIN BD11, and mouse islets. Other in vitro systems further examined EEEC’s glucose-lowering properties. Obese rats fed a high-fat-fed diet (HFF) were selected for in vivo evaluation, and phytoconstituents were detected via RP-HPLC followed by LC-MS. EEEC induced insulin secretion in a concentration-dependent manner with modulatory effects, similar to 1 µM glucagon-like peptide 1 (GLP-1), which were partly declined in the presence of Ca^2+-^channel blocker (Verapamil), K_ATP_-channel opener (Diazoxide), and Ca^2+^ chelation. The insulin secretory effects of EEEC were augmented by isobutyl methylxanthine (IBMX), which persisted in the context of tolbutamide or a depolarizing concentration of KCl. EEEC enhanced insulin action in 3T3-L1 cells and reduced glucose absorption, and protein glycation in vitro. In HFF rats, it improved glucose tolerance and plasma insulin, attenuated plasma DPP-IV, and induced active GLP-1 (7-36) levels in circulation. Rhodomyrtosone B, Quercetin-3-O-β-D-glucopyranoside, rhodomyrtosone E, and quercitroside were identified as possible phytoconstituents that may be responsible for EEEC effects. Thus, these findings revealed that *E. citriodora* could be used as an adjunct nutritional supplement to manage type 2 diabetes.

## 1. Introduction

Diabetes mellitus (DM) is a progressive group of metabolic illnesses that has become a major global concern [1] since it is currently the 7th leading cause of mortality [2]. Nearly 10.5% of the global population is suffering from DM [3] and approximately 90% of these individuals are type 2 diabetes mellitus (T2DM) patients [4]. The major pathophysiology of T2DM includes insulin resistance, obesity, chronic inflammation, oxidative stress, and mitochondrial dysfunction. Other than these genetic factors, lifestyle habits are also attributed to diabetes prevalence and incidence [5]. Obesity, generally known as an overabundance of body fat that adversely affects human health, is often measured in BMI (body mass index). The presence of nonesterified fatty acids (NEFAs) released from adipose tissue in obese patients may raise concerns of insulin resistance and β-cell dysfunction that leads to type 2 diabetes [6]. Obesity may also increase the risk of hypertension and dyslipidaemia that lead to cardiovascular disease (CVD) (such as coronary artery disease, angina, atrial fibrillation, myocardial infarction, and sudden cardiac arrest) in type 2 diabetes patients [7]. However, there is no complete cure for DM, except for symptomatic treatment [8].

One of the current treatments for DM is DPP-IV inhibitors, which work by suppressing the activity of the dipeptidyl peptidase IV (DPP-IV) enzyme. DPP-IV enzyme is responsible for inactivating the incretin hormones, Glucose-dependent insulinotropic polypeptide (GIP) and glucagon-like peptide-1 (GLP-1) by cleaving in N-terminal, resulting in GIP (3–42) and GLP-1 (9–36) [9]. These hormones are released from the intestine following nutrient intake and bind to specific receptors in pancreatic β-cells. This binding enhances the cyclic adenosine monophosphate (cAMP) pathway and thus stimulates insulin release from the clonal β-cells. Hence, the incretin hormones play a vital role in regulating post-prandial hyperglycaemia [10]. As it is known, the DPP-IV enzyme degrades the incretin hormones and reduces the life span of GLP-1 and GIP [9]. Therefore, DPP-IV inhibitors are an effective measure for controlling diabetes mellitus. However, DPP-IV inhibitors and other synthetic current therapeutics are raising a wide concern of adverse effects such as gastrointestinal disturbances, weight gain, diarrhea, renal failure, and hypoglycemia [11]. As a result, alternative medicines, particularly plant-based medicines, are frequently desired [5].

Several reports support that herbal medicine might be one of the successful approaches to maintaining T2DM globally [12], without provoking adverse effects or secondary complications. Phytochemicals including tannins, flavonoids, vitamin C, and E are potent antioxidants and have been indicated to ameliorate pancreatic β-cell functions, enhancing insulin action and glucose transport and modulating glucagon-like peptide-1 (GLP-1) homeostasis [13,14]. Several phytochemicals such as rutin, isoquercitrin, and catechin have been observed to improve insulin secretion via DPP-IV inhibition, thus protecting the active forms of the incretin hormones, GIP (1–42) and GLP-1 (7–36), from degradation [15].

*Eucalyptus (*Family*- Myrtaceae)* is a large genus of evergreen aromatic trees. This genus is native to Australia, India, Bangladesh, and the neighboring islands. Among other species of this genus, *Eucalyptus citriodora* Hook, commonly called the lemon- or citron-scented gum [16], is a well-known medicinal plant with bluish green leaves [17] and composed of volatile (essential oil and sterols) [16] and non-volatile chemical constituents (triterpenes, tannins, flavonoids, anthocyanins, and phenolic compounds) [18]. Traditionally, the leaf extract of *E. citriodora* was used as a remedy to treat various diseases, including diabetes, whooping cough, liver and gallbladder disorders, ulcer, neuralgia, stomatitis, pain, gonorrhea, rheumatism, and gastrointestinal disturbances [18]. Furthermore, volatile essential oils isolated from its leaves were found to have ethnomedicinal use against cold, fever, and bronchitis along with suppurative and general respiratory tract infections such as asthma and chronic obstructive pulmonary disease (COPD) [19]. *Eucalyptus citriodora* has a wide range of pharmacological indications with low or no toxicity reports [18], and some of these properties include antidiabetic, antifungal, antispasmodic, antibacterial, antiseptic, anti-inflammatory, analgesic, and diuretic activity, inhibition of bone resorption, and natural repellency [20,21]. Similarly, hot water extract of *E. citriodora* augmented insulin release from pancreatic β-cells, improved glucose tolerance and β-cell function, and reduced plasma DPP-IV concentration in high-fat fed (HFF) obese rats [22]. The present study aims to evaluate the basic mechanism of action of the antidiabetic activity exerted by ethanol extract of *E. citriodora* leaves both in vitro and in vivo.

## 2. Materials and Methods

### 2.1. Collection and Preparation of Plant Extracts

The leaves of *E. citriodora* were obtained from Bandarban, a tropical and hilly area of Bangladesh. The collection was performed during monsoon season when the average temperature was between 26–30 °C. A taxonomist identified the plant’s identity and assigned the accession number 43755. The leaves of *E. citriodora* were properly cleaned and air-dried in an oven at 40 °C before being extracted with ethanol. Plant Powder (200 g) was dissolved in 1 L of 80% (*v*/*v*) ethanol and kept on shaker at 900 g for 48–72 h at room temperature. The mixture was filtered using Whatman no. 1 filter paper, and the extract was allowed to dry under decreased pressure at <40 °C. The oily filtrate was vacuum-dried (Savant Speed vac; New York, NY, USA) using a rotary evaporator machine (Bibby RE-200, Sterilin Ltd., Newport, UK) and the ultimate product, a gummy, semi-solid crude extract of *E. citriodora* was achieved (~6 g) and stored at 4 °C for experimental studies [23]. EEEC was not soluble in water, therefore the extract was dissolved in 60 μL/10 mL dimethyl sulfoxide (0.6% DMSO) and was not toxic to the cells for further investigations.

### 2.2. In Vitro Insulin-Releasing Studies 

The insulin-releasing effects of ethanol extract of *E. citriodora* (EEEC) was examined in clonal BRIN-BD11 cells (ECACC 10033003) [24] and isolated mouse islets [25]. Islets were isolated from the pancreas of mice via collagenase P obtained from *Clostridium histolyticum* and cultured for 24–48 h in an incubator at 37 °C [26]. Leaf extract of *E. citriodora* with or without known insulin secretagogues at various glucose concentrations (1.1, 5.6, or 16.7 mM) were incubated at 37 °C for 20 and 60 min, respectively [25]. The samples were collected, centrifuged, and stored at –20 °C for insulin radioimmunoassay [27]. For the measurement of insulin content in the islet cells, an acid-ethanol extraction method was employed [26].

### 2.3. Membrane Potential and Intracellular Calcium ([Ca^2+^]_i_)

A FLIPR Membrane Potential and Calcium Assay Kit (Molecular Devices, Sunnyvale, CA, USA) were used to determine the influence of EEEC on membrane potential and intracellular calcium [Ca^2+^] in BRIN-BD11 cells [28]. At first, microplates with ninety-six wells were used to seed BRIN-BD11 cells and allowed to stand for 18 h at 37 °C. After the removal of the medium, cells in 100 μL KRB buffer solution were incubated at 37 °C for 10 min. The positive controls were a depolarizing concentration of 30 mM KCl and 10 mM alanine. The changes in signal intensity caused by EEEC were observed in a fluorometer [5].

### 2.4. Cellular Glucose Uptake

The insulin action of EEEC and its impact on the cellular glucose uptake were evaluated using 3T3L1 differentiated cells as discussed previously [29]. The cells were first incubated with 50 µL EEEC (200 µg/mL) at 37 °C for 30 min in the presence/absence of 100 nM insulin followed by the addition of 2-NBDG (50 nM). The resultant mixture was allowed to stand for 5 min and then rinsed with ice-cold PBS. The slides were fitted with coverslips and the corners were tightly sealed with nail polish. A microscope (10× magnification) was used to capture images of the coverslips and the fluorescence intensity was assessed as previously stated [21].

### 2.5. Glycation of Insulin

The impact of EEEC on in vitro glycation of insulin was measured as described [30]. D-Glucose (246·5 mM), Human insulin (1 mg/mL), sodium phosphate buffer (10 Mm, pH 7.4) and NaBH3CN (0.0853 g/mL), with (treatment)/without (control) plant extract were mixed to create a volume of 1 mL. The resultant mixture was incubated at 37 °C for 24 h and the reaction was ended by adding 30 µL of 0.5 M acetic acid. Aminoguanidine, a known inhibitor of protein glycation, was used as the positive control, and glycated and non-glycated insulin were detected via RP-HPLC [31].

### 2.6. DPP-IV Enzyme Activity In Vitro

DPP-IV enzyme activity in vitro was analyzed using a fluorometer following the processes stated earlier [32]. The test reagents, DPP-IV (8 mU/mL) enzyme and Gly-Pro-AMC (200 µM) with (treatment) or without (control) treatment, were incubated in 96-well black-walled, clear-bottomed microplates (Greiner). The fluorescence intensity was calculated using a Flex Station 3 (Molecular Devices, Sunnyvale, CA, USA) consisting of a 2.5 nm slit width with excitation and emission at 370 nm and 440 nm, respectively. The drug sitagliptin was considered a positive control [22].

### 2.7. Starch Digestion

An assay for in vitro starch digestion under the influence of EEEC was performed on the previous study [33]. A mixture of starch solution (2 mg/mL; 100 mg in 50 mL water), with (treatment) or without (control) treatment and heat stable α-amylase (40 µL of 0.01%) (from *Bacillus leicheniformis*, Sigma-Aldrich, St. Louis, MO, USA) were incubated at 80 °C for 20 min. The resultant diluted mixture was again incubated with amyloglucosidase (30 µL of 0.1%, Sigma-Aldrich) from Rhizopus mold at 60 °C for 30 min. Samples were collected and stored at 4 °C until further analysis for the glucose release using liquid GOD/PAP method (Randox GL 2623) [22]. As a positive control, α-glucosidase inhibitor, acarbose was used.

### 2.8. Glucose Diffusion In Vitro

A cellulose ester dialysis tube (CEDT) filled with 2 mL volume of 0.9% NaCl and 220 mM glucose in the presence (treatment) or absence (control) of plant extract was used to assess the in vitro glucose diffusion and absorption [34]. CEDT was placed inside a 50 mL centrifuge tube (Orange Scientific, Orange, CA, USA) consisting of 0.9% NaCl (45 mL) solution after being tightly sealed from both ends. Samples were removed after 24 h at 37 °C from the orbital shaker to analyze the glucose diffusion [22].

### 2.9. Animals

**Figure 1:**
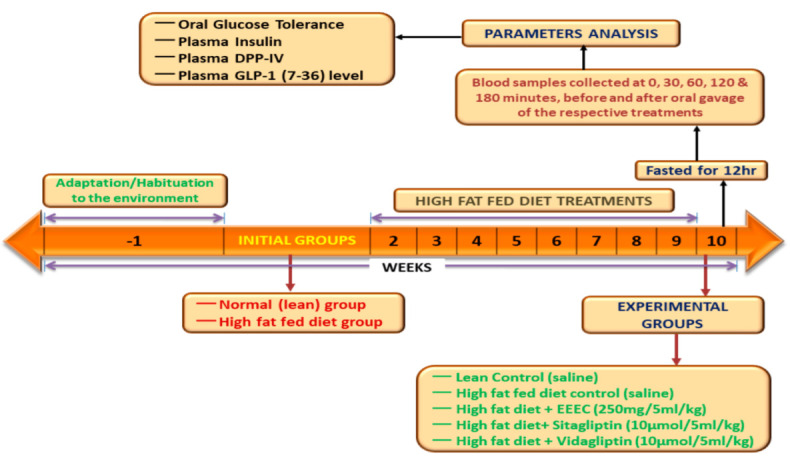
Schematic diagram represents the experimental design for animal studies.

At about 6–8 weeks before the start of the experiments, 8–9-week-old Sprague–Dawley male rats (Envigo UK, approximately 200–250 g) were fed a high-fat diet [20% protein, 45% fat, and 35% carbohydrate: 26.15 KJ/g total energy percent (Special Diet Service, Essex, UK)]. The age-matched rats were fed a standard rodent diet [10% fat, 30% protein, and 60% carbohydrate making 12.99 KJ/g total energy (Trouw Nutrition, Cheshire, UK)] and these rats were used as controls. Animals were housed under a maintained temperature and humidity (25 ± 0.5 °C and 65–70%) conditions. Animal housing had a 12 h automatic light on-off facility that ensured a day-night circadian rhythm. All experiments were approved by the Animal Welfare and Ethical Review Board (AWERB) (14 May 2018) at Ulster University and were conducted under the PIL1822 and PPL 2804 UK Home Office Animal project/personal license numbers, which were authorized on 6 May 2016 and 5 February 2017, respectively. The experiments were performed in compliance with the UK Act 1986 and EU Directive 2010/63EU. Measures were performed to ensure that no animals would be harmed during the duration of the study. Figure 1 illustrates a summary of the experimental design for animal studies.

### 2.10. Oral Glucose Tolerance Test

High-fat-fed rats (380–400 g body wt.) were used to assess the effects of EEEC on blood glucose. All the blood samples were collected at specific times after oral administration of glucose (18 mmol/kg body weight) with and without EEEC (250 mg/5 mL/kg), as indicated in Figure 4B. Plasma was obtained by centrifuging the blood for 5 min at 12,000 rpm at 4 °C and then storing it at 20 °C for insulin assay. Blood glucose was measured using an Ascencia Contour glucose meter (Bayer, Newbury, UK), and insulin was assessed by dextran-charcoal radioimmunoassay [34].

### 2.11. DPP-IV Enzyme Activity

The effects of EEEC on the DPP-IV enzyme were measured in plasma using a fluorometric assay, as reported previously [25]. After oral gavage of EEEC (250 mg/5 mL/kg), DPP-IV inhibitors sitagliptin (10 mol/5 mL/kg) and vildagliptin (10 mol/5 mL/kg), or saline control, blood samples were collected from rats fed a high-fat diet at certain times, as indicated in Figure 4D. Using a GLP-1 (Active) ELISA Kit (EGLP-35K, Merck Millipore, Dorset, UK), active GLP-1 (7–36) was measured in plasma samples collected at 30 min.

### 2.12. Purification of Crude Extract

The possible active phytoconstituents from the EEEC leaf were isolated using the techniques described previously [34]. Initially, crude leaf extract was dissolved in 0.12 percent (*v*/*v*; TFA/water) for further evaluation by RP-HPLC, where separation of the components was performed based on their affinity. The filtered extract was injected into the stainless-steel column of the RP-HPLC, which was then equilibrated with 0.12 percent (*v*/*v*; TFA/water) at a flow rate of 1.0 mL/min. Acetonitrile was utilized as an eluent and was retained at a gradient ratio of 20% over 10 min and 70% over a 40 min cycle. The peak fractions were collected at 254 nm, and the individual peak retention times on the HPLC were recorded [25].

### 2.13. Mass Spectroscopy

Molecular weights of peak fractions of EEEC from the RP-HPLC were determined by means of liquid chromatography-mass spectrometry (LC-MS) through electrospray ionization mass spectrometry (ESI-MS) method [25]. Peak fractions of the samples were separated and identified via Spectra System LC (Thermo Separation Products) using a Kinetex 5m F5 LC column (150 × 4.6 mm, Phenomenex) and a UV detection system (220–256 nm) following the detailed methodology discussed previously [34].

### 2.14. Statistical Analysis

Analysis and interpretation of the raw data were performed using Graph Pad prism 5. Unpaired Student’s *t*-test (nonparametric, with two-tailed *p* values) in addition to one-way ANOVA with Bonferroni post hoc tests were applied for the analysis of the data and the values were stated as Mean ± SEM with a hypothetical significance limit of *p* < 0.05 [22,25].

## 3. Results

### 3.1. EEEC and Insulin Release

**Figure 2:**
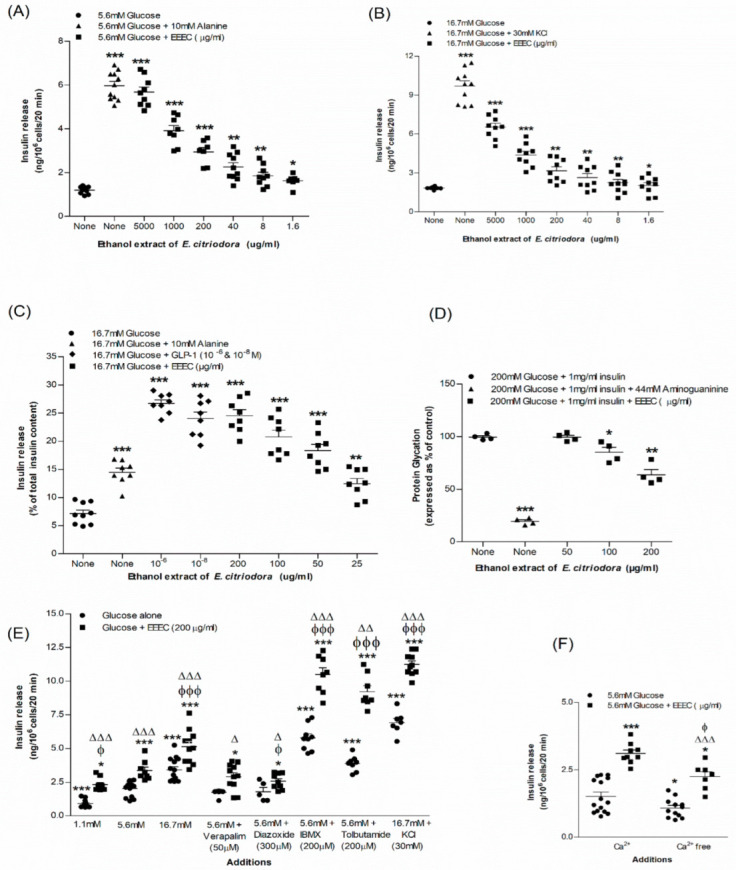
Effects of EEEC on insulin secretion from (**A**,**B**) clonal pancreatic β-cells (BRIN-BD11) and (**C**) islets of Langerhans, (**D**) glycation of protein, (**E**) secretion of insulin with known stimulators or inhibitors and (**F**) plus or minus extracellular calcium from clonal β-cells. Values *n* = 8 and 4 for insulin secretion and *n* = 3 for glycation of protein are mean ± SEM. * *p* < 0.05, ** *p* < 0.01 and *** *p* < 0.001 compared to control. ^ϕ^ *p* < 0.05 and ^ϕϕϕ^ *p* < 0.001 compared to 5.6 mM glucose with EEEC. ^Δ^ *p* < 0.05, ^ΔΔ^ *p* < 0.01 and ^ΔΔΔ^ *p* < 0.001 compared to respective incubation without EEEC. EEEC, Ethanol extract of *E. citriodora*.

The basal rate of insulin release from BRIN-BD11 cells was 1.20 ± 0.05 ng/10^6^ cells/20 min at 5.6 mM glucose. The rate increased to 5.97 ± 0.19 ng/10^6^ cells/20 min (*p* < 0.001; Figure 2A*; n* = 8) in the presence of a positive control, alanine (10 mM). The effect of increasing concentrations of EEEC on insulin release from BRIN-BD11 cells are demonstrated in Figure 2A,B. EEEC produced 1.63 ± 0.10 to 5.69 ± 0.22 ng/10^6^ cells/20 min (*p* < 0.05–0.001; Figure 2A; *n* = 8) insulin following a dose-dependent manner (1.6–5000 μg/mL) at 5.6 mM glucose. At 16.7 mM glucose, the basal concentration of insulin release from clonal pancreatic β cell line, BRIN-BD11 was 1.84 ± 0.03 ng/10^6^ cells/20 min which reached 9.70 ± 0.40 ng/10^6^ cells/20 min (*p* < 0.001; Figure 2B; *n* = 8) with a depolarized concentration of KCl (30 mM). EEEC significantly (*p* < 0.001) stimulated insulin release at 16.7 mM glucose from 2.03 ± 0.22 to 6.53 ± 0.29 ng/10^6^ cells/20 min at 1.6-5000 μg/mL (*p* < 0.05–0.001; Figure 2B; *n* = 8). However, no remarkable effect of EEEC on lactate dehydrogenase release was observed over the concentration range of 1.6–200 μg/mL. EEEC produced a substantial increase in insulin secretion from isolated mouse islets at 16.7 mM glucose (Figure 2C). EEEC portrayed a significant increase in insulin secretion at ≥25 μg/mL (*p* < 0.001; Figure 2C). Insulin secretion by EEEC was comparable to that induced by GLP-1 (10^−6^ & 10^−8^ M) when tested at 200 µg/mL concentration (*p* < 0.001; Figure 2C).

### 3.2. EEEC and Known Modulators of Insulin Release, Inhibitors and Free Ca^2+^ Conditions

EEEC at a concentration of 200 µg/mL was incubated with known stimulators or inhibitors in BRIN-BD11 cells to determine the insulin response (Figure 2E). At 5.6 mM glucose, K^+^ channel activator, diazoxide (300 µM), and L-type voltage-dependent Ca^2+^ channel blocker, verapamil (50 μM), lowered (15–24%) the insulin-releasing properties of EEEC by interfering with intracellular Ca^2+^ influx activity (Figure 2E). Additionally, KCl (30 mM), a depolarizing stimulus, increased insulin release of EEEC by 1.7-fold (*p* < 0.001; Figure 2E). Moreover, isobutyl methylxanthine (IBMX) (*p* < 0.001; Figure 2E) and tolbutamide (Figure 2E; *p* < 0.001) also increased the action of the EEEC in inducing insulin release whereas, in the absence of extracellular calcium, it was inhibited by 34% (Figure 2F).

### 3.3. EEEC and Membrane Depolarization and [Ca^2+^]_i_

**Figure 3:**
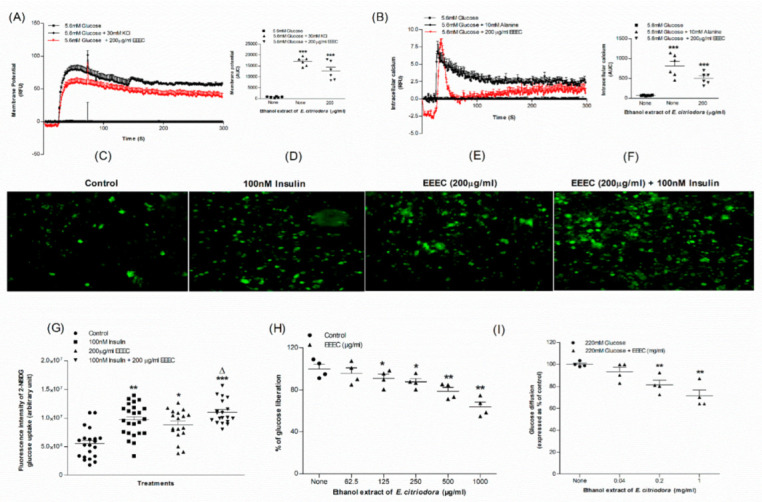
Effects of EEEC on (**A**) membrane potential and (**B**) intracellular calcium in clonal pancreatic β cell (BRIN BD11) and, (**C**–**G**) glucose uptake, (**H**) starch digestion and (**I**) glucose diffusion in vitro. Changes of fluorescence intensity in differentiated 3T3L1 adipocyte incubated with EEEC (**E**) minus or (**F**) plus 100 nM insulin. The ×10 magnification was used to take the images. Values *n* = 6 for membrane potential and intracellular calcium, *n* = 4 for uptake of glucose, digestion of starch and diffusion of glucose are mean ± SEM. * *p* < 0.05, ** *p* < 0.01, and *** *p* < 0.001 compared to control. ^Δ^ *p* < 0.05 compared to insulin alone.

The membrane depolarization of BRIN-BD11 cells was significant *(p <* 0.001) with 30 mM KCl. EEEC also depolarized the membrane (*p* < 0.001; Figure 3A) in the presence of 5.6 mM glucose. Both Alanine (*p* < 0.001) and EEEC (*p* < 0.001) resulted in a substantial elevation in intracellular calcium concentration at 5.6 mM glucose (Figure 3B).

### 3.4. EEEC and Glycation of Insulin

EEEC demonstrated a significant inhibitory effect on insulin glycation. It produced a 14% inhibition at its lowest concentration of 100 µg/mL, while it produced a maximum 34% inhibition at 200 µg/mL (*p* < 0.001; Figure 2D). Aminoguanidine, (44 mM) used as a positive control, inhibited insulin glycation by 80.5% (*p* < 0.001; Figure 2D).

### 3.5. EEEC and Glucose Uptake and Insulin Action

EEEC was studied for effects on glucose uptake by 3T3L1 differentiated adipocyte cells using a glucose analogue, 2-NBDG (2-(N-(7-Nitrobenz-2-oxa-1,3-diazol-4-yl) Amino)-2-Deoxyglucose) fluorescent hexose (Figure 3C–G). EEEC significantly increased glucose uptake *(p <* 0.05; Figure 3G). Insulin at a concentration of 100 nM exacerbated this stimulatory effect *(p <* 0.001; Figure 3G). Glucose uptake decreased to 34%, with EEEC alone, from about 45% recorded in the presence of insulin (Figure 3G).

### 3.6. EEEC and Starch Digestion

EEEC at ≥125 µg/mL concentration reduced (*p* < 0.05) digestion of starch by 9% (Figure 3H) whereas at a higher concentration (1000 µg/mL), the reduction of glucose liberation from starch was 34%. The positive control, Acarbose, considerably (*p* < 0.001) inhibited enzymatic glucose liberation from starch by 85% (Data not provided).

### 3.7. EEEC and Glucose Diffusion In Vitro

After 24 h of incubation with glucose, it was observed that EEEC (mg/mL) significantly inhibited glucose diffusion and absorption in a dose-dependent manner (*p* < 0.01; Figure 3I). EEEC demonstrated a 28% inhibitory effect (*p* < 0.01) at 1 mg/mL (Figure 3I).

### 3.8. EEEC and DPP-IV Enzyme Activity In Vitro

**Figure 4:**
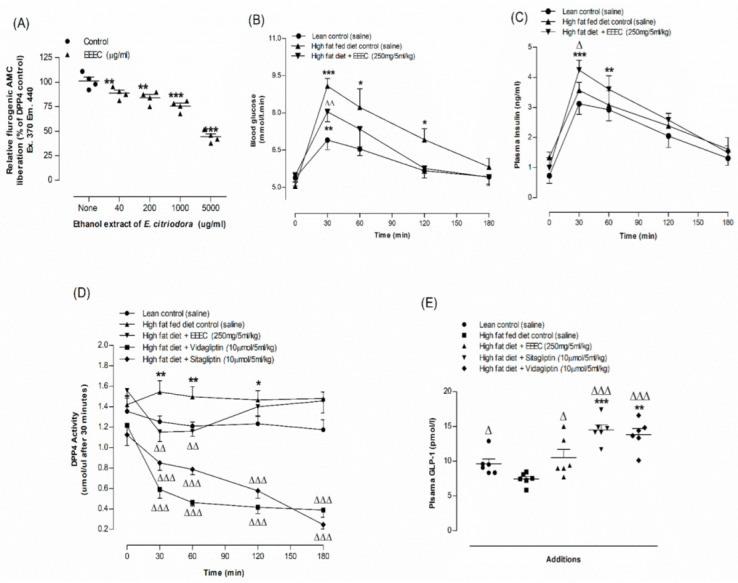
Effects of EEEC on (**A**) DPP-IV enzyme in vitro, (**B**) glucose tolerance, (**C**) plasma insulin, (**D**) DPP-IV and (**E**) plasma active GLP-1 (7–36) in high-fat-fed rats. In vivo parameters were evaluated before and after oral gavage of glucose alone (18 mmol/kg body weight, control) or with EEEC (250 mg/5 mL/kg body weight), sitagliptin and vidagliptin (both at 10 μmol /5 mL/kg, body weight). Plasma active GLP-1 (7–36) levels was assayed at 30 min after treatments. Values *n* = 4 for in vitro DPP-IV enzyme activity and *n* = 6 for in vivo parameters are mean ± SEM. * *p* <0.05, ** *p* <0.01 and *** *p* < 0.001 compared to lean control and ^Δ^ *p* < 0.05, ^ΔΔ^ *p* < 0.01 and ^ΔΔΔ^ *p* < 0.001 compared to high-fat-fed diet control rats.

Results for in vitro DPP-IV enzyme inhibitory effect of EEEC are portrayed in Figure 4A. EEEC inhibited the DPP-IV enzyme by 9–52% in a concentration-dependent manner (40–5000 µg/mL) (*p* < 0.01; *p* < 0.001; Figure 4A). An established drug named Sitagliptin (10 µM) has been found to inhibit the enzymatic liberation of AMC from the DPP-IV substrate, Gly-Pro-AMC by 97.5% (Data not provided).

### 3.9. EEEC and Oral Glucose Tolerance and Plasma Insulin, DPP-IV and Active GLP-1 (7-36) Levels

An oral dose of EEEC (250 mg/5 mL/kg; b.w.) improved glucose tolerance, plasma insulin concentration, and reduced plasma DPP-IV substantially (*p* < 0.05–0.01; Figure 4B-D). Sitagliptin and vildagliptin (10 μmol/5 mL/kg), considered as positive controls, depicted significant (*p* < 0.001; Figure 4D) reduction (70–73%, Figure 4D) in DPP-IV enzyme activity. At 30 min after oral administration of EEEC, active GLP-1 (7–36) concentrations in plasma increased by 28% (*p* < 0.05–0.01; Figure 4E) and with sitagliptin and vidagliptin, it increased to 86–92% (*p* < 0.001; Figure 4E).

### 3.10. Identification of Purified Extract

**Figure 5:**
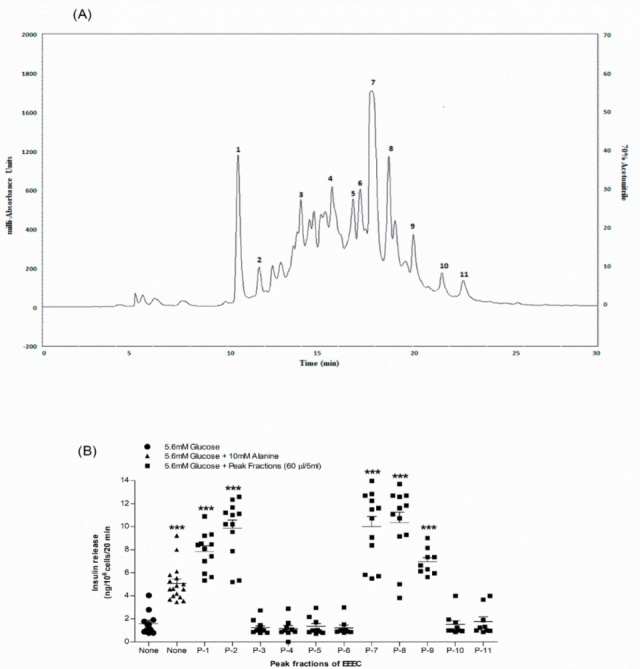
Representative (**A**) HPLC profile and (**B**) insulin-releasing effects of peak fractions (1,2,7-9) of EEEC. Crude extract was chromatographed at a flow rate of 1.0 mL/min on a (10 × 250 mm) semi-preparative 5 μm C-18 column (Phenomenex, UK). Using linear gradients of acetonitrile (0–20% up to 10 min, 20–70% up to 40 min), the concentration of the eluting solvent was increased. Compounds were detected by measurement of absorbance at 254 nm. Peak fractions 1, 2 and 7–9 were collected and insulin-releasing activity assessed using BRIN-BD11 cells. Values *n* = 8 for insulin release are mean ± SEM. *** *p* < 0.001 compared to control.

**Figure 6:**
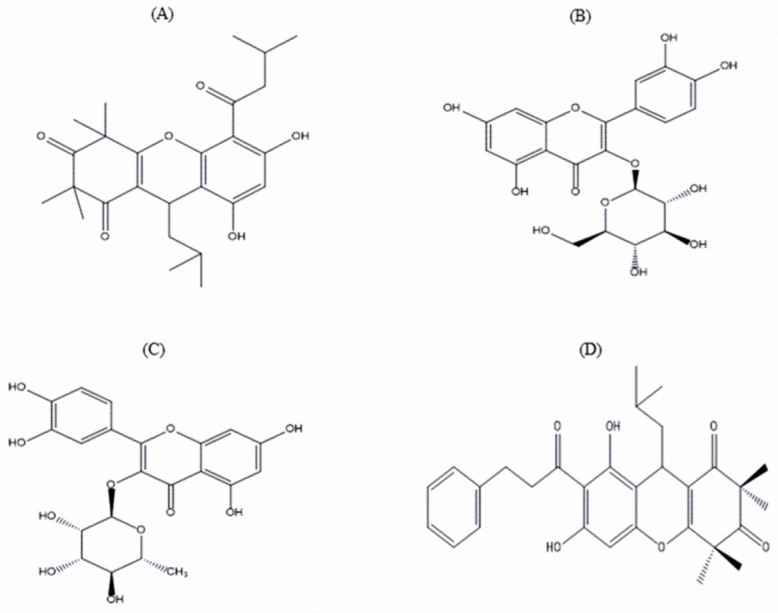
Chemical structure of possible phytoconstituents extracted from the EEEC. Chemical structures of (**A**) Rhodomyrtosone B, (**B**) Quercetin-3-O-β-D-glucopyranoside, (**C**) Quercitroside and (**D**) Rhodomyrtosone E with their corresponding molecular formulae: C_26_H_34_O_6,_ C_21_H_20_O_12_, C_21_H_20_O_11_, and C_30_H_34_O_6._

The pharmacologically possible active molecules characterized from EEEC bark are listed in Table 1. We collected eleven major peaks from the EEEC via RP-HPLC (Figure 5A) and then were assayed for insulin secretory properties using clonal pancreatic β-cells (BRIN BD11). Peak fractions P-1, P-2, P-7, P-8, and P-9 greatly (*p* < 0.001; Figure 5B) produced insulin secretion, however P-1 and P-2 were associated with cell toxicity. Alanine was used as a standard control (*p* < 0.001; Figure 5B). Peak fractions of concern were further investigated using LC-MS (Table 1). The molecular masses of P-1, P-2, P-7, P-8, and P-9 were 442.1, 476.7, 464.2, 490.9, and 447.9 Da, respectively. Figure 6A–D depicts the chemical structures of anticipated compounds based on their molecular mass.

## 4. Discussion

Diabetes mellitus (DM), a metabolic group of disorders, is currently the fastest growing chronic disease around the world and a severe threat to public health worldwide [34,35]. DM is a result of the absolute or relative deficiency of insulin secretion and/or insulin resistance and is diagnosed as a high blood glucose level that can lead to a variety of secondary acute or chronic complications [35]. For decades, medicinal plants and their phytochemicals have been found to have significant pharmacological and biological benefits. Therefore, they have been increasingly used as replacement therapy for synthetic drugs. Although *E. citriodora* has anti-hyperglycemic activities, the exact mechanism of action is elusive [22]. Therefore, the present study was designed to investigate the insulin secretory effects of EEEC on isolated mouse islets and BRIN-BD11 cells, including its other hypoglycemic properties.

EEEC significantly increased insulin secretion from clonal pancreatic β-cells and isolated islets in a dose-dependent manner. To understand the molecular mechanism of insulin secretion more accurately, the effects of a non-toxic dose of EEEC were evaluated in the presence or absence of insulin increasing/reducing modulators. A class of oral hypoglycemic medications known as sulfonylureas works by blocking K_ATP_ channels and depolarizing the plasma membrane, which stimulates Ca^2+^ entry via activating voltage-dependent calcium channels [36]. In the presence of both a K_ATP_ channel blocker (tolbutamide) and membrane depolarization with 30 mM KCl, EEEC stimulated insulin secretion. Thus, this indicates that the EEEC has the capability to enhance insulin secretion via several pathways, such as direct impacts on exocytosis, PI_3_ (phosphatidylinositol pathway), adenylate cyclase, or cAMP pathway [37]. Diazoxide partially decreased the insulin-releasing activity of the EEEC, suggesting the involvement of K_ATP_ channel blockage, which is further supported using verapamil, a voltage-dependent Ca^2+^ channel blocker, which suppressed insulin secretory properties of EEEC [35]. EEEC promoted membrane depolarization and raised intracellular calcium levels in BRIN BD11 cells. In the presence of the cAMP phosphodiesterase inhibitor IBMX, EEEC substantially increased insulin secretion, indicating the involvement of the cAMP pathway [22]. EEEC can cause an increase in cAMP in lung tissues, which inhibits the reproduction of bronchial smooth muscle cells and enhances airway relaxation, and this has been proven to be useful in the treatment of asthma [38,39].

Insulin predominantly modulates postprandial glucose, and anomalies in the signal transduction pathway in skeletal muscle and adipose tissue, such as decreased GLUT4 translocation, are a major contributor to insulin resistance [40,41]. Therefore, the impact of EEEC on glucose uptake in differentiated 3T3L1 adipocyte cells was examined. It was observed that the EEEC significantly enhances glucose transport with or without insulin. According to previous studies, flavonoids and other polyphenols enhance glucose uptake and insulin action by modulating AMP-activated protein kinase (AMPK), phosphoinositide 3-kinase (PI3K), and mitogen-activated protein kinase (MAPK) activity [42,43,44]. *E. citriodora* contains polyphenols such as flavonoids which may activate signalling pathways to promote glucose transport in adipocytes in the presence or absence of insulin [42,45].

Protein glycation is a physiological mechanism that is crucial in the pathogenesis of diabetes. Chronic hyperglycemia leads to non-enzymatic glycation of proteins such as albumin, fibrinogen, immunoglobulins, and collagen [46]. The increased production of free radicals leads to the formation of advanced glycation end products (AGE). AGEs can accumulate intracellularly and disrupt the function of plasma proteins and enhance oxidative stress, and this accelerates the severity of diabetic complications including retinopathy, neuropathy, and nephropathy [46,47]. In this study, EEEC substantially inhibited protein glycation in a concentration-dependent manner. Recent studies also reported that *E. citriodora* protects β-cells damaging by inhibiting protein glycation via free radical scavenging [47]. Another study with *E. citriodora* indicated antioxidant properties, and it might help to overcome oxidative stress-induced protein damage [20,48,49]. Plants containing flavonoids, such as procyanidin, epicatechin, and rhodomyrtosone E, have also been indicated to have anti-glycation properties in recent studies [8,22,23].

Postprandial hyperglycaemia is considered to be a risk factor for CVD, and thus treating postprandial may prevent secondary cardiovascular complications in type 2 diabetic patients [50]. Inhibition of α-amylase and α-glucosidase activities are an essential strategy for managing postprandial hyperglycemia and the inhibition of these enzymes might be beneficial to type 2 diabetes patients with impaired insulinotropic response [49]. In the present studies, EEEC significantly inhibited starch hydrolysis in a concentration-dependent manner. Furthermore, flavonoids and other phytochemicals have been observed to suppress α-amylase and α-glucosidase activities [51]. Therefore, the present findings indicate that *E. citriodora* contains polyphenols such as isoquercitrin, gallic acid, quercitrin and betulinic acid, which may have the potential to restrict glucose absorption [52,53].

The molecular mass and concentration of soluble dietary fibers play a vital role in glucose-lowering actions of herbs [38,54]. To mimic the gut and investigate the effects of EEEC on glucose diffusion, an in vitro dialysis method was performed. EEEC exhibited concentration-dependent suppressing effects on glucose transport across a dialysis membrane into an external solution. It has been demonstrated that dietary supplementation with *E. citriodora* reduces hyperglycemia in STZ-induced diabetic rats, which is believed to be due to the inhibition of glucose absorption from the gut [33]. *E. citriodora* has also been observed to lower blood glucose levels in alloxan-induced diabetic rats, suggesting similar effects to the anti-diabetic drug glibenclamide [18].

It is observed that a high-fat diet induces obesity and metabolic abnormalities such as insulin resistance, and the propensity to develop type 2 diabetes in healthy Sprague–Dawley rats [55]. In an acute in vivo study, EEEC improved glucose tolerance and plasma insulin levels in rats fed a high-fat diet. Recent studies also reported that hot water extract of *E. citriodora* ameliorates glucose tolerance, plasma insulin and β-cell function in high-fat-fed rats [22].

The unique signaling mechanism of DPP-IV inhibitors, which involves delaying the degradation of incretin hormones (GLP-1 and GIP), causing decrease in glucagon release, and improving insulin secretion, has demonstrated a significant impact on the treatment of type 2 diabetes [56]. DPP-IV enzyme deactivates incretin hormones via N-terminal cleavage, resulting in GLP-1 (9–36) and GIP (3–42). This disrupts the regulation of insulin release and increases blood glucose levels [30]. Therefore, suppression of DPP-IV enzyme activity may play a vital role in delaying the deactivation of GLP-1 and GIP, which may boost insulin secretion and positively regulate blood glucose levels [57]. In this investigation, EEEC inhibited the DPP-IV enzyme activity in vitro concentration-dependently. In vivo studies on rats fed a high-fat diet revealed a significant reduction of plasma DPP-IV enzyme activity and a substantial increase in active GLP-1 (7–36) concentration. Previous studies indicated that natural sources, such as plants and their phytochemicals including isoquercitrin, rutin, and gallic acid, have the potential to inhibit the DPP-IV enzyme [34,58,59]. These findings imply that the insulin-releasing and glucose-lowering effects of EEEC may be partly attributable to DPP-IV enzyme suppression.

RP-HPLC and LC-MS techniques were employed to explore the possible active phytoconstituents of EEEC. Based on our initial screening, we have detected several peak fractions among which P-1, P-2, P-7, P-8, and P-9 increased insulin release from BRIN BD11 cells that have been hypothesized to be active molecules contributing to antihyperglycaemic action of EEEC. The molecular mass of peak fractions P1, P-7, P-8, and P-9 were corresponded to rhodomyrtosone B [60,61], quercetin-3-O-β-D-glucopyranoside [62], rhodomyrtosone E [5], and quercitroside [63]. The results agree with previous findings of *E. citriodora* containing isoquercitrin, rhodomyrtosone E, and quercitrin [21,64,65]. No previous reports have proven rhodomyrtosone B as a phytomolecule of *E. citriodora*, while others have extracted rhodomyrtosone B from *Rhodomyrtus tomentosa,* a plant known to have blood glucose lowering, antioxidant, and antihyperlipidemic properties [60,66]. Recent findings also depicted that quercetin-3-O-β-D-glucopyranoside improves glucose tolerance and increases plasma GLP-1 levels by reducing DPP-4 enzyme activity in STZ-induced diabetic rats [67]. Additionally, it has been observed that quercitrin attenuates oxidative stress by scavenging free radicals and reducing lipid peroxidation, thus increasing insulin production from pancreatic β-cells in STZ-induced diabetic rats [68]. Another compound, rhodomyrtosone E, enhanced GLUT4 translocation in L6 skeletal muscle cells by AMPK pathway [69]. All these predicted compounds may be responsible for these activities of EEEC. Further investigations using other parameters such as NMR are warranted to elucidate the chemical structure of the potential phytoconstituents.

## 5. Conclusions

The current investigations indicated that EEEC has the potential to enhance insulin secretion from clonal pancreatic β-cells. EEEC substantially regulated hyperglycemia by suppressing glucose absorption, protein glycation, and DPP-IV enzyme activity in vitro, respectively. EEEC ameliorated glucose tolerance and plasma insulin and abated DPP-IV enzyme activity in rats fed a high-fat diet, thus possibly increasing the half-life of incretin hormone (GLP-1 and GIP). Insulin secretory and glucose-lowering effects of EEEC are partly due to the presence of anticipated phytoconstituents, including quercetin-3-O-β-D-glucopyranoside, rhodomyrtosone B and E, and quercitroside. Additional studies including chronic studies in animal models are needed to completely explore the role of EEEC and its phytomolecules in the retardation of diabetes mellitus progression. Further extensive research on humans is required to determine the effective dose in the management of T2DM. Thus, *E. citriodora* may be useful as a dietary supplement for managing diabetes mellitus and its complications.

## Figures and Tables

**Table 1:** Molecular mass of peak samples of EEEC leaves obtained from the preparative RP-HPLC via LC-MS analysis.

Peak Samples	Retention Time (min)	Theoretical Molecular Weight (Da)	Found Molecular Weight (Da)	Predicted Compounds
P_1_	10.5	442.5	442.1	Rhodomyrtosone B
P_2_	12	-	476.7	Unknown
P_7_	18	464.4	464.2	Quercetin-3-O-β-D-glucopyranoside
P_8_	19	-	490.9	Rhodomyrtosone E
P_9_	20.5	448.4	447.9	Quercitroside

Peaks were separated using a Kinetex 5 µm F5 LC column ((150 × 4.6 mm^2^) (Phenomenex) on a Spectra System LC. The mass-to-charge ratio (m/z) was analyzed against peak intensity. Fragments of “peak-1 (P_1_), peak-2 (P_2_), peak-7 (P_7_), peak-8 (P_8_), and peak-9 (P_9_)” with retention time 10.5, 12, 18, 19, and 20.5 min determined the molecular weight of unknown compounds with m/z 442.1, 476.7, 464.2, 490.9, and 447.9 Da, respectively.

## Data Availability

The corresponding author can provide the data presented in this study upon request. Due to restrictions, the data are not available to the public.

## Abbreviations

AGE	Advanced glycation end-products
AMPK	AMP-activated protein kinase
BMI	Body mass index
CEDT	Cellulose ester dialysis tube
cAMP	Cyclic adenosine monophosphate.
COPD	Chronic obstructive pulmonary disease
CVD	Cardiovascular diseases
DM	Diabetes Mellitus
DPP-IV	Dipeptidyl peptidase IV
EEEC	Ethanol extract of *Eucalyptus citriodora*
GIP	Glucose-dependent insulinotropic polypeptide
GLP-1	Glucagon-like peptide-1
GLUT4	Glucose transporter type 4
HFF	High-fat-fed
IBMX	3-isobutyl-1-methylxanthine
KCl	Potassium chloride
LC-MS	Liquid chromatography–mass spectrometry
MAPK	Mitogen-activated protein kinase
NEFA	Non-esterified fatty acids
NMR	Nuclear magnetic resonance
PI3K	Phosphoinositide 3-kinase (PI3K)
RP-HPLC	Reverse-phase high performance liquid chromatography
T2DM	Type 2 Diabetes Mellitus
